# BMAL1/FOXA2-induced rhythmic fluctuations in IL-6 contribute to nocturnal asthma attacks

**DOI:** 10.3389/fimmu.2022.947067

**Published:** 2022-11-25

**Authors:** Lingling Tang, Li Liu, Xianhong Sun, Po Hu, Hui Zhang, Bohan Wang, Xiaona Zhang, Jinjin Jiang, Xia Zhao, Xiaolu Shi

**Affiliations:** ^1^ Jiangsu Province Hospital of Chinese Medicine, Affiliated Hospital of Nanjing University of Chinese Medicine, Nanjing, Jiangsu, China; ^2^ Department of Central lab, Affiliated Hospital of Nanjing University of Chinese Medicine, Nanjing, Jiangsu, China; ^3^ Department of Respiration, Changzhou Hospital of Traditional Chinese Medicine, Changzhou, Jiangsu, China; ^4^ Department of Respiration, Nanjing Hospital of Chinese Medicine Affiliated to Nanjing University of Chinese Medicine, Nanjing, Jiangsu, China; ^5^ Jiangsu Key Laboratory of Pediatric Respiratory Disease, Institute of Pediatrics, Affiliated Hospital of Nanjing University of Chinese Medicine, Nanjing, Jiangsu, China

**Keywords:** asthma, nocturnal symptom, circadian, inflammatory, airway epithelial cell

## Abstract

The circadian clock is closely associated with inflammatory reactions. Increased inflammatory cytokine levels have been detected in the airways of nocturnal asthma. However, the mechanisms that contribute to the nocturnal increase in inflammatory responses and the relationship with circadian clock remain unknown.

**Methods:**

Inflammatory cytokine levels were measured in asthma patients with and without nocturnal symptoms. Allergic airway disease was induced in mice by ovalbumin (OVA), and different periods of light/dark cycles were used to induce circadian rhythm disorders. Serum shock was used to stimulate the rhythmic expression in human bronchial epidermal cells (16HBE). The expression and oscillation of circadian clock genes and inflammatory cytokines in 16HBE cells subjected to brain and muscle ARNT-like protein-1 (BMAL1) and Forkhead Box A2 (FOXA2) knockdown and treatment with a FOXA2 overexpression plasmid were assessed.

**Results:**

Serum IL-6 was found to be significantly higher in asthmatic patients with nocturnal symptoms than those without nocturnal symptoms. The OVA-induced asthma model with a circadian rhythm disorder and 16HBE cells treated with serum shock showed an increase in IL-6 levels and a negative correlation with BMAL1 and FOXA2. The knockdown of BMAL1 resulted in a lower correlation between IL-6 and other rhythm clock genes. Furthermore, knockdown of the BMAL1 and FOXA2 in 16HBE cells reduced the expression and rhythmic fluctuations of IL-6.

**Conclusions:**

Our findings suggest that there are increased IL-6 levels in nocturnal asthma resulting from inhibition of the BMAL1/FOXA2 signalling pathway in airway epithelial cells.

## Introduction

Asthma is a heterogeneous disease of the airways involving a variety of cells and cellular components. Its clinical presentation includes recurrent wheezing, shortness of breath, chest tightness, coughs that vary over time and in intensity, and variable airflow limitations (1). One asthma symptom that worsens at night is nocturnal asthma, which affects nearly 30–70% of patients (2, 3). Nocturnal asthma also appears to be associated with an increased risk of mortality as 50–68% of asthma-related deaths and respiratory arrest occurred at night (4, 5). Previous studies have demonstrated a circadian variation in the fractional exhaled nitric oxide (FeNO) and lung function in nocturnal asthma (6–8), which may be triggered by inflammations in the airways at night. FeNO has emerged as an important biomarker of T helper 2 (Th2)-mediated airway inflammation in asthma (9). Nocturnal asthma is therefore associated with a significant increase in airway inflammation at night.

The circadian clock (approximately 24 h) is a collection of intrinsic, endogenous physiological oscillators that are found in almost all lung tissue cells (10). The circadian clock system plays a crucial role in the regulation of nearly all physiological activities. Disordered circadian rhythms therefore have severe consequences on human health. The disruption of circadian clock machinery leads to the dysregulation of the inflammatory response (11). Night shift workers with disrupted circadian rhythms eventually experience autoimmunity (a mistimed rise in IL-6), which compromises host defence mechanisms (12). The genes involved in circadian rhythms include period (PER1-2), cryptochrome (CRY1-2), nuclear receptor subfamily 1, group D, member 1 (NR1D1), CLOCK, and BMAL1. Mice lacking BMAL1 gene expression in myeloid cells have been found to exhibit markedly increased inflammation when challenged with OVA (13). An improved understanding of the correlation between circadian and inflammatory cytokines may therefore allow for strategies to be devised for the prevention, treatment, and recovery from asthma.

In this study, we detected inflammatory cytokines in asthma patients with and without nocturnal symptoms and found that inflammatory factors, such as IL-6, were increased in the peripheral blood of patients with nocturnal asthma. We further explored the relationship between circadian clock genes and inflammatory cytokines in a disordered circadian rhythm using OVA-challenged mice and cellular serum shock models. In addition, we discovered a negative regulatory effect of FOXA2 on IL-6 and determined that FOXA2 is involved in the circadian clock genes that co-regulate the rhythmic fluctuations of IL-6.

## Materials and methods

### Human samples

This study was approved by the Ethics Committee of the Affiliated Hospital of Nanjing University of Chinese Medicine (approval number 2019NL-024-02). All patients provided informed consent.

We retrospectively analysed the medical records of patients with asthma who were admitted to the Affiliated Hospital of Nanjing University of Chinese Medicine (Nanjing, Jiangsu, China) from November 2019 to December 2021. The inclusion criteria for patients with asthma included a clinical diagnosis of asthma according to the Global Initiative for Asthma Guidelines (2021) (14).

Patients with combined serious primary diseases or missing data were excluded from the analysis. The data that were assessed included baseline clinical data, pulmonary function tests, comorbidities, immunoglobulin E (IgE), routine blood tests, blood biochemistry, and cytokine measurements. Treatment decisions were made by the designated physician throughout the study period and were not influenced by the protocol for the study.

Nocturnal asthma, which is defined by symptoms that include coughing, wheezing, and dyspnoea, worsens at night and disturbs sleep (15). Blood samples from all patients were therefore collected in the morning between 4–5 A.M. Blood was collected from asthma patients with nocturnal symptoms on the days of attacks. Serum samples were frozen and stored at –80 °C.

### Animals

All of the protocols involving animal use were approved by the Animal Ethics Committee of the Affiliated Hospital of Nanjing University of Traditional Chinese Medicine (NO:2021 DW-07-02). Thirty-six BALB/c mice (female, 18-22g, aged 6–8 weeks, Laboratory Animal Center of Jiangsu Academy of Traditional Chinese Medicine) were acclimatised in a specific pathogen-free room (temperature: 22°C ± 2°C, humidity: 55% ± 5%) with free access to food (irradiation feed) and water (sterilized). The animal experiments were performed in compliance with the ARRIVE guidelines.

### OVA-induced mouse asthma model

The OVA-induced asthma model was established based on a previously described method (16). In brief, all mice were randomly assigned into two groups (control group n=6; asthma group, n=30). mice received intraperitoneal (i.p.) administrations of 100 µg of OVA complexed with 20 µg of alum (Sigma-Aldrich, St Louis, US) or saline on days 0 and 14. The mice were further challenged with 1 mg of OVA or saline intranasally (i.n.) on days 14, 25, 26, and 27 (**Figure S1**).

### Circadian rhythm disorder mouse model

The circadian rhythm disorder mouse model used in this study was performed as previously described in other studies (17–19). On day 14 of asthma model construction, asthma group mice were randomly separated into five cohorts after sensitisation and challenge, with six mice per group. The control group (N) and normal asthma group (A) were housed with 12 h dark/12 h light (DL12) cycles. The mice from the other asthma group were individually housed with 3.5 h dark/3.5 h light (DL3.5) cycles, 24 h dark/24 h light (DL24) cycles, all-day darkness (DD), and all-daylight (LL) until day 28. Darkness began at 6:00 p.m. Lavage fluid, sera, and lung tissue were collected at 6:00 p.m. on day 28 (**Figure S1**).

### Lung tissue analysis

Mice were anesthetised with pentobarbital (50 mg/kg). Blood plasma was collected by the abdominal aortic method. The cervical muscles and blood vessels were bluntly separated. The trachaeas were exposed by surgically opening the neck regions with minimum incisions. The lungs were lavaged using a trachaeal tube with 500 μL of chilled phosphate buffered saline (Servicebio, Wuhan, China) and a syringe while gently massaging the lung area to ensure adequate rinsing. This procedure was repeated three times. The collected bronchoalveolar lavage fluid (BALF) was immediately centrifuged at 500×g for 5 min at 4°C. The supernatant was stored at 4°C for further analysis. Flow cytometry was used to analyse the different cell types in the BALF (20). After collecting the BALF, the thoracic cavities of the mice were cut open. The inferior lobes of the left lungs in each group were removed and fixed in 4% paraformaldehyde aqueous solution (Servicebio) for the immunohistochemistry and haematoxylin-eosin (HE) staining procedures. The right lungs were immediately snap-frozen in liquid nitrogen and stored at –80°C until the total RNA and protein extractions for real-time quantitative polymerase chain reaction (RT-qPCR) and Western blot could occur.

### Cell culture

Human bronchial epithelial cells (16HBE) and human embryonic kidney cells (293T) were obtained from the cell bank of the Chinese Academy of Sciences (Shanghai, China). 16HBE cells were cultured with RPMI 1640 media (Gibco, New York, USA) and 293T cells cultured with Dulbeccos’s modified Eagle’s medium (DMEM; Gibco), both containing 10% foetal bovine serum (FBS; Evergreen, Hangzhou, China), 100 mg/L penicillin, and 100 mg/L streptomycin (Gibco) in a 5% CO_2_ incubator at 37°C. When cells grew to 70%-80% confluence, they were passaged using trypsin-ethylenediaminetetraacetic acid (Gibco).

### Serum shock

Serum shock-induced synchronisation was performed based on a previously described method (21, 22). In brief, 16HBE cells were seeded in medium plates with 1×10^6^ cells/plate and cultured in serum-free RPMI 1640 medium (Biological Industries, Haemek, Israel) for 24 h prior to the experiments. On the day of the experiments, the culture media were replaced with RPMI 1640 medium containing 50% horse serum (Yuanye, Shanghai, China) for 2 h. The media were then replaced with serum-free RPMI 1640. The cells were harvested and assayed at different time points (0, 4, 8, 12, 16, 20, 24, 28, 32, 36, 40, and 44 h) following serum shock and labelled as ZT0–ZT44, respectively.

### Cell transfection

The 16HBE cells were seeded in 6-well plates. Follow-up experiments were performed when cell confluence reached 80%. Silent shRNAs (Hanbio, Shanghai, China) were transfected using the LipoFiter transfection reagent (Hanbio) in serum-free media. The cells were then transferred to 10% FBS-containing RPMI 1640 medium 6 h after transfection. After culturing for another 24 h, the positive populations were selected using Blasticidin S (Beyotime, Shanghai, China). The target sequences of the shRNAs have been listed in **Table S1**. The FOXA2 overexpression plasmid was purchased from iGene Biotechnology Co., Ltd. The 16HBE cells were transfected with the plasmid using Lipofectamine 8000 (Beyotime) according to the manufacturer’s instructions. Twenty-four hours after transfection, the medium was replaced with RPMI 1640 medium containing 10% FBS, and the cells were cultured for another 24 h. Gene silencing and overexpression were tested at the mRNA and protein levels.

### ELISA

The human and mice blood samples were collected and centrifuged at 3000 rpm for 10 min at 4°C to obtain serum samples. The culture supernatant was harvested after serum shock at different time points. After centrifugation, the supernatant was taken and stored at –80°C. The serum levels of OVA-IgE were measured using an anti-OVA IgE ELISA kit (Cayman, Michigan, US). Interferon-α (INF-α), IL-2, IL-4, IL-5, IL-6, IL-8, IL-10, IL-12, IL-13, IL-17, IL-1β, and tumour necrosis factor (TNF)-α were quantified using ELISA kits (Multi Sciences, Hangzhou, China) following the manufacturer’s instructions.

### Immunohistochemistry

The mouse lung specimens were dehydrated, embedded in paraffin, and cut into 4-µm-thick sections. After deparaffinisation and rehydration, the sections were incubated with 3% hydrogen peroxide for 10 min to inactivate the endogenous peroxidase activity. After antigen retrieval, membrane permeabilisation was induced using 0.1% Triton X-100 in PBS for 20 min. The sections were blocked with 10% goat serum for 1 h. The slides were then incubated over night at 4°C with the primary antibodies (BMAL1, 1:200, Proteintech, 14268-1 and IL-6, 1:200, Affinity, DF6087). The secondary antibody consisting of HPR-labelled goat anti-rabbit IgG (1:200, Proteintech, S0001) was added and incubated for 1 h. Proteins were visualised with DAB (brown). Nuclei were counterstained with haematoxylin (blue).

### Hematoxylin-eosin staining

The dewaxing and antigen retrieval steps were the same as those used for immunohistochemistry. First, the sections were stained with haematoxylin solution for 5 min and rinsed with tap water for 5 min. The sections were then differentiated in 1% acid alcohol for 30 s and washed again with tap water. In the bluing step, the sections were immersed in saturated lithium carbonate solution for 1 min and rinsed with tap water. Finally, the sections were counterstained with eosin Y solution for 3 min.

### Western blot

The lung tissues were cut into small pieces. An appropriate amount of RIPA lysis buffer (Beyotime) containing a protease and phosphatase inhibitor mixture (Bimake, Houston, TX, US) and grinding steel balls were added and placed in a grinder to produce the homogenate. The homogenate was centrifuged at 12000× g for 20 min at 4°C. Supernatants were obtained after centrifugation. Protein concentrations were measured using the BCA protein detection kit (Beyotime). The whole cell lysates were added to a sodium dodecyl sulfate loading buffer, boiled for 10 min, and stored at –80°C. The proteins were isolated by sodium dodecyl sulfate-polyacrylamide gel electrophoresis and transferred to PVDF membranes using a membrane transfer apparatus. The membranes were blocked with 5% skimmed milk at room temperature. The corresponding primary antibodies (BMAL1, 1:1000, proteintech, 14268-1; IL-6, 1:1000, affinity, DF6087; FOXA2, 1:1000, abcam-ab108422) were then added. The membranes were incubated overnight at 4°C. After washing with Tris-buffered saline with 0.1% Tween^®^ 20 Detergent (TBST) on the second day, the corresponding HRP-labelled goat anti-rabbit IgG antibody (1:5000, Proteintech, S0001) was added and incubated for 2 h. Finally, the protein bands were visualised following colouration with an enhanced chemiluminescence (ECL) reagent. Using GAPDH as the internal reference, the grey values of the target protein bands and the ratio of the grey values to the internal reference protein were analysed using ImageJ software.

### Quantitative reverse transcription PCR

Partial lung lobes of mice and 16HBE cells were collected, and total RNA was extracted using an RNA Extraction Kit (Vazyme, Nanjing, China). cDNA was synthesised using a reverse transcription kit and stored at –20°C. The primer sequences for each gene have been listed in **Table S2**. The qPCR was performed using an ABI 7500 fast fluorescent quantitative PCR instrument. The thermocycling conditions were as follows: pre-denaturation at 95°C for 5 min, followed by 95°C for 10 s and 60°C for 30 s for 40 consecutive cycles. Using β-actin as an internal reference, the data were processed according to the calculation method of the relative expression amount = 2 ^−△△ Ct^ to compare the changes in the mRNA expression levels of the samples in each group.

### Luciferase reporter assays

The 293T and 16HBE cells were seeded into 96-well plates. They were transfected with IL-6 luciferase reporters, the pRL-TK vector, and the FOXA2 overexpression plasmid or blank pcDNA using LipoFiter3.0 (Hanbio). Twenty-four hours later, the medium was removed, and the cells were collected for luciferase activity measurements using the Dual-Lumi™ II Luciferase Reporter Gene Assay Kit (Beyotime) according to the manufacturer’s instructions. Firefly luciferase values were normalised to the Renilla luciferase values.

### Statistical analysis

For the statistical analyses, we used SPSS21.0 (IBM Corp., Armonk, NY, USA). Sex, allergic history, corticosteroid therapy, and comorbidities comprised the qualitative data that were analysed using Fisher’s exact test. Shapiro-Wilk and Levene’s tests were used to confirm the normal distribution and homogeneity of variances of the quantitative data. Independent sample *t*-tests and a one-way analysis of variance (ANOVA) were used to compare the groups after the relevant conditions had been met. Tukey’s multiple comparisons analysis method was used to compare the experimental and control groups. The Kruskal-Wallis test was performed on datasets with unequal variances. Pearson’s correlation coefficient was used to determine the strength of the link between inflammatory cytokines and rhythm clock genes.

The Benjamini-Hochberg algorithm was used to adjust the *p*-values for the false discovery rate. All statistical tests were two-sided, and *p* < 0.05 was considered statistically significant

### Role of Funders

The Funders did not have any role in study design, data collection, data analyses, interpretation, or writing of the report.

## Results

### Serum IL-6 expression is elevated in asthma patients with nocturnal symptoms

Between November 2019 and December 2021, 45 patients were diagnosed with asthma and admitted to our hospital. Patients without sufficient data (n = 7) and those with combined serious primary diseases (n = 1) were excluded. Eighteen asthma patients with nocturnal symptoms and 18 patients without nocturnal symptoms were included in the study. The baseline clinical, demographic, and biological characteristics of the patients (age, sex, BMI, heart rate, breathing rate, temperature, allergic history, corticosteroid therapy, pulmonary function tests, comorbidities, IgE, routine blood tests, and blood biochemistry) were similar between the groups, as shown in **Table 1**. The IL-6 levels for the asthma patients with nocturnal symptoms were markedly higher than those for patients without nocturnal symptoms (7.45 ± 6.57 vs. 2.81 ± 1.49 pg/mL, *p* = 0.002). In addition, the TNF-α levels were higher in the asthma patients with nocturnal symptoms (3.39 ± 1.90 vs. 2.09 ± 1.80 pg/ml, *p* = 0.043), but the between-group differences in the inflammatory cytokines, except IL-6 (adjusted *p* = 0.022), were not statistically significant after correction using the Benjamini–Hochberg method (**Table 2**).

**Table 1:** Demographic and clinical characteristics of the patients.

Variable	Asthma patients with nocturnal symptoms (n = 18)	Asthma patients without nocturnal symptoms (n = 18)	*p-*value
Age (y), mean(range)	59.61 ± 14.58 (24–87)	61.50 ± 11.46 (47–83)	0.464
Sex			
Male (%)	10 (55.56%)	4 (22.22%)	0.086
Female (%)	8 (44.44%)	14 (77.78%)	
BMI (kg/m^2^), mean (range)	23.55 ± 3.20 (16.65-26.67)	24.63 ± 3.54 (19.77-33.3)	0.248
Heart rate (bpm), mean (range)	86.94 ± 16.01 (56–119)	82.94 ± 6.73 (76–100)	0.495
Breathing rate (br/min), mean (range)	19.17 ± 1.79 (16–22)	18.44 ± 1.42 (16–20)	0.188
Temperature (°C), mean (range)	36.35 ± 0.38 (35.8-37.4)	36.42 ± 0.29 (36.0-37.2)	0.406
Allergic history (%)	12 (66.67%)	9 (50.00%)	0.500
Corticosteroids therapy (%)	14 (77.78%)	9 (50.00%)	0.164
FEV1 (%predicted), mean (range)	79.88 ± 26.42 (24.5-107.4)	68.48 ± 17.83 (31.1-102.9)	0.138
FEV1/FVC, mean (range)	74.73 ± 15.71 (36.48-98.36)	68.49 ± 15.52 (39.0-98.82)	0.239
Comorbidities			
Hypertension (%)	5 (27.78%)	6 (33.33%)	>0.999
COPD (%)	6 (33.33%)	4 (22.22%)	0.711
Coronary heart disease (%)	3 (16.67%)	1 (5.56%)	0.603
IgE (IU/mL), mean (range)	242.28 ± 367.93 (10–1421)	304.44 ± 397.42 (8–1410)	0.656
Routine blood test			
WBC (10^9^/L), mean (range)	8.24 ± 2.64 (5.01-11.81)	6.96 ± 2.18 (4.6-11.74)	0.121
RBC (10^12^/L), mean (range)	4.39 ± 0.55 (3.53-5.43)	4.18 ± 0.50 (3.43-5.38)	0.244
HGB (g/L), mean (range)	132.17 ± 15.82 (98–162)	122.61 ± 14.68 (84–152)	0.069
PLT (10^9^/L), mean (range)	201.11 ± 50.35 (72–300)	237.28 ± 108.31 (133–541)	0.944
EO%, mean (range)	2.79 ± 4.38 (0–17)	7.19 ± 12.63 (0-53.5)	0.171
LYM%, mean (range)	20.01 ± 9.56 (4.4-36.1)	25.79 ± 9.67 (3.4-46.8)	0.080
NEUT%, mean (range)	69.89 ± 13.02 (51.9-94.2)	61.86 ± 11.96 (38.8-92.8)	0.062
Blood biochemistry			
AST (U/L), mean (range)	16.89 ± 5.98 (9–28)	15.89 ± 4.43 (9–25)	0.572
ALT (U/L), mean (range)	17.39 ± 8.49 (10–46)	15.33 ± 5.54 (8–27)	0.610
TP (g/L), mean (range)	65.75 ± 6.48 (54.93-78.41)	64.45 ± 5.93 (55.46-82.02)	0.628
ALB (g/L), mean (range)	40.79 ± 4.58 (30.4-47.8)	40.76 ± 3.91 (32.3-49.8)	0.978
GLOB (g/L), mean (range)	24.36 ± 5.10 (15.7-37.1)	23.00 ± 6.28 (18.1-45)	0.145
ALP (U/L), mean (range)	74.86 ± 28.04 (14–126)	70.89 ± 16.62 (42–106)	0.609
CREA (μmol/L), mean (range)	65.72 ± 14.45 (47.9-99.9)	60.13 ± 16.67 (32.3-101.3)	0.290
UREA (mmol/L), mean (range)	5.68 ± 2.00 (3.06-11.32)	5.16 ± 1.40 (2.43-7.92)	0.370

Data are presented as the mean ± SEM or percentages and were compared using Fisher’s exact test, Kruskal-Wallis test, or independent sample t-tests. No significant differences were observed between the groups.

ALB, albumin; ALP, alkaline phosphatase; ALT, alanine aminotransferase; AST, aspartate amino transferase; BMI, body mass index; COPD, chronic obstructive pulmonary disease; CREA, creatinine; EO, eosinophils; FEV1, forced expiratory volume in 1 second; FEV1/FVC, forced expiratory volume in one second/forced vital capacity; GOLB, globulin; HGB, hemoglobin count; IgE, immunoglobulin E; LYM, lymphocytes; NEUT, neutrophils; PLT, platelet; RBC, red blood cell count; TP, total protein; WBC, white blood cell count.

**Table 2:** Inflammatory cytokines of asthma patients with or without nocturnal symptoms.

Variable	Asthma patients with nocturnal symptoms (n = 18)	Asthma patients without nocturnal symptoms (n = 18)	*p-*value	adjust *p-*value
IL-5 (pg/mL), mean (range)	4.25 ± 9.56 (0.19-39.85)	5.09 ± 8.62 (0.2-30.5)	0.079	0.290
IFN-α (pg/mL), mean (range)	2.20 ± 1.90 (0.4-7.12)	1.60 ± 0.77 (0.3-3.74)	0.420	0.770
IL-2 (pg/mL), mean (range)	1.51 ± 0.36 (0.99-2.20)	1.45 ± 0.57 (0.59-3.01)	0.749	0.915
IL-6 (pg/mL), mean (range)	7.45 ± 6.57 (1.35-28.65)	2.81 ± 1.49 (0.66-6.53)	0.002**	0.022*
IL-1β (pg/mL), mean (range)	9.31 ± 6.22 (0.94-17.08)	7.86 ± 10.87 (0.47-42.6)	0.126	0.347
IL-10 (pg/mL), mean (range)	1.52 ± 0.71 (0.79-3.81)	1.65 ± 0.92 (0.45-4.15)	0.622	0.887
IL-8 (pg/mL), mean (range)	3.43 ± 3.43 (0.09-11.77)	3.21 ± 4.74 (0.14-19.83)	0.645	0.887
IL-17 (pg/mL), mean (range)	1.72 ± 0.74 (0.49-2.82)	1.42 ± 0.48 (0.31-1.99)	0.156	0.343
IL-4 (pg/mL), mean (range)	1.28 ± 0.59 (0.27-2.33)	1.26 ± 0.43 (0.35-1.67)	0.761	0.941
IL-12 (pg/mL), mean (range)	1.21 ± 0.67 (0.26-2.66)	1.23 ± 0.71 (0.01-3.01)	0.941	0.941
TNF-α (pg/mL), mean (range)	3.39 ± 1.90 (0.78-7.05)	2.09 ± 1.80 (0.58-6.8)	0.043*	0.237

Data are presented as mean values ± SEM, the two groups were compared with Kruskal-Wallis test or independent samples t-tests. The Benjamini-Hochberg algorithm was used to adjust p-values. *p < 0.05, **p < 0.01 compared with asthma patients without nocturnal symptoms.

### Serum IL-6 expression is elevated in asthmatic mouse models with circadian rhythm disorders

**Figure 1:**
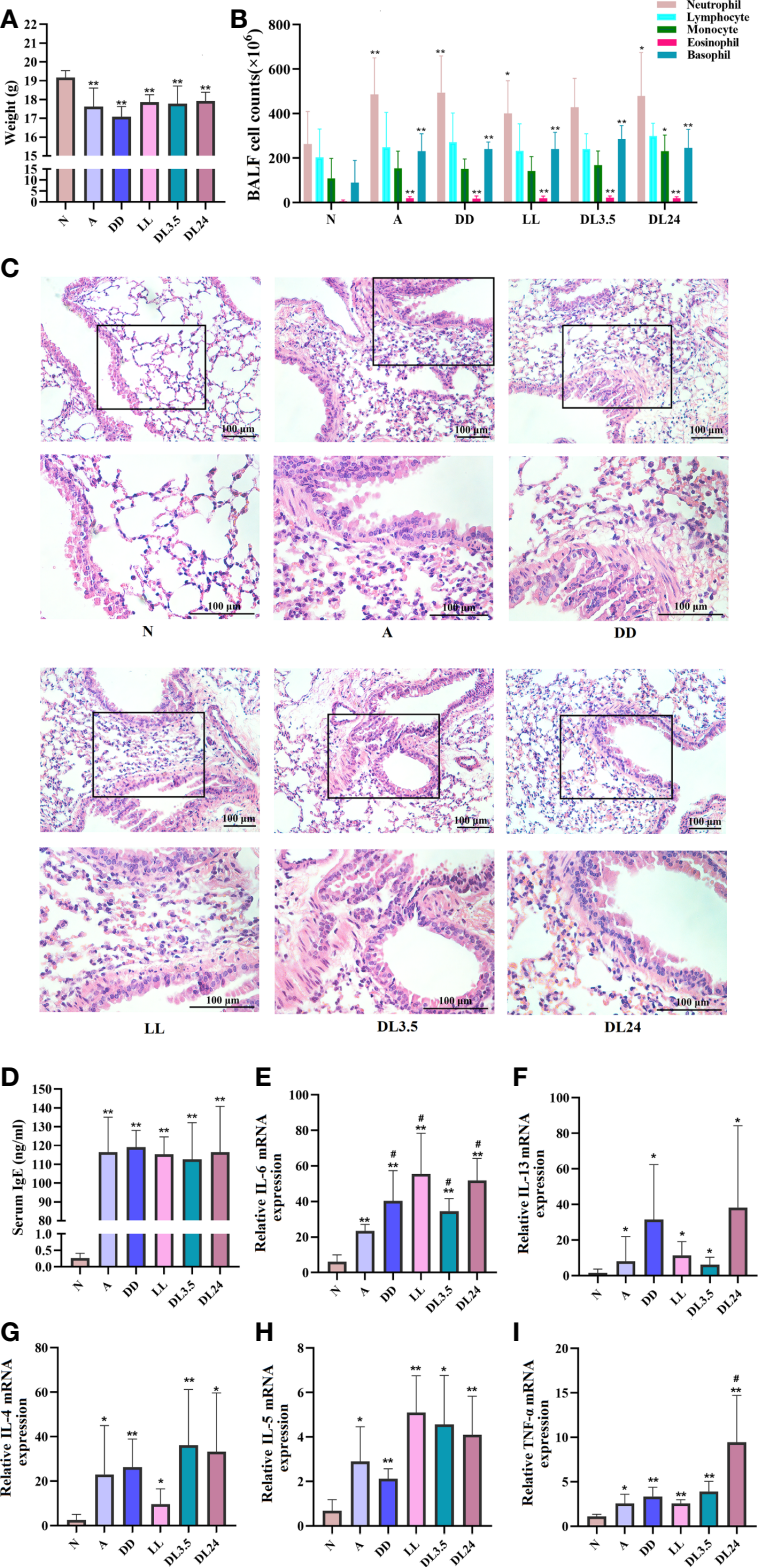
Effects of light-induced disturbances of rhythm on airway inflammation in the lung tissues of OVA-challenged mice. **(A)** Body weight variations at the end of the experiment in each group of mice. **(B)** Flow cytometric analysis of cell populations in the BALF of mice. **(C)** H&E staining of sections from OVA-challenged mouse lung tissue. Magnification: 100× (upper panel) and 200× (lower panel). Scale bar: 100 μm. **(D)** ELISA analysis of IgE levels in serum. **(E–I)** RT-qPCR analysis of the mRNA levels of inflammatory cytokines (IL-6, IL-13, IL-4, IL-5, and TNF-a) in the lung tissue. N=control group; A=OVA group; DD=OVA + all-day darkness group; LL= OVA + all-daylight; DL3.5= OVA + 3.5 h dark/3.5 h light group; DL24= OVA + 24 h dark/24 h light group. All data are presented as the mean ± standard error of the mean (SEM) of 6 mice per group, *p < 0.05, **p < 0.01, compared with the control group. ^#^ *p* < 0.05. One-way ANOVA with Tukey’s multiple comparison analysis method.

The nocturnal asthma symptoms of coughing, wheezing, and dyspnoea are accompanied by circadian variations in airway inflammation, and the circadian clock may be responsible for this variability (23, 24). Therefore, we used different periods of light/dark cycles to induce circadian rhythm disorders in asthmatic mice and investigated the correlation between inflammatory cytokines and rhythm clock genes. The asthmatic mice lost weight (**Figure 1A**), had a significantly higher number of inflammatory cells in the BALF (**Figure 1B**), a marked increase in the infiltration of inflammatory cells and airway smooth muscle thickness (**Figure 1C**), and increased OVA-specific serum IgE levels (**Figure 1D**). However, these results did not differ from those of the asthmatic mice with circadian rhythm disorders. This indicates that different periods of light/dark cycles do not increase lung pathological changes in asthmatic mice. We further analysed the inflammatory cytokines in the sera of the mice and found that 24 h dark/24 h light cycles increased TNF-α levels (**Figure 1I**). Four different periods of light/dark cycles all increased IL-6 levels in the sera of asthmatic mice (**Figure 1E**), which was similar to the inflammatory cytokine results of the asthmatic patients. We did not observe significant differences in IL-4, IL-5, and IL-13 levels after the asthmatic mice had undergone different periods of light/dark cycles (**Figures 1F–H**).

### Potential relationship between IL-6 and circadian clock genes

**Figure 2:**
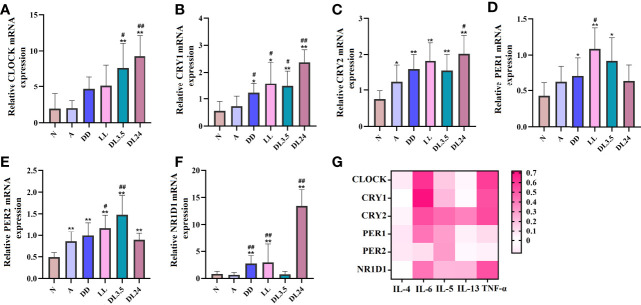
Association between circadian clock genes and inflammatory cytokines in mouse lung tissues. **(A–F)** RT-qPCR analysis of the mRNA levels of inflammatory cytokines (CLOCK, CRY1, CRY2, PER1, PER2, and NR1D1) in lung tissue. **(G)** Heat map of the matrix coefficient correlation of each circadian clock gene and inflammatory cytokines. The stronger Pearson’s correlation coefficients (r) are represented in red. N=control group; A=OVA group; DD=OVA + all-day darkness group; LL= OVA + all-daylight; DL3.5= OVA + 3.5 h dark/3.5 h light group; DL24= OVA + 24 h dark/24 h light group; BLAF= bronchoalveolar lavage fluid. All data are presented as the mean ± SEM of 6 mice per group, *p < 0.05, **p < 0.01, compared with the control group. ^#^ *p* < 0.05, ^##^ *p* < 0.01 compared with the OVA group. One-way ANOVA with Tukey’s multiple comparison analysis method, Pearson’s correlation coefficient.

**Figure 3:**
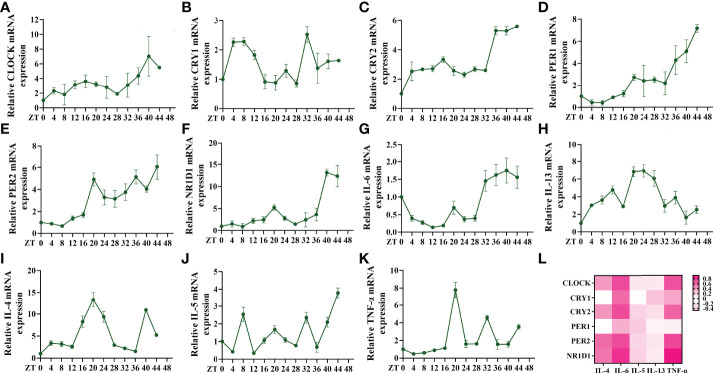
Serum shock induces circadian clock gene and inflammatory cytokine expression and association in 16HBE cells. **(A–F)** RT-qPCR analysis of the mRNA levels of circadian clock genes (CLOCK, CRY1, CRY2, PER1, PER2, and NR1D1) in 16HBE cells in response to serum shock synchronisation. **(G–K)** RT-qPCR analysis of the mRNA levels of inflammatory cytokines (IL-6, IL-13, IL-4, IL-5, and TNF-a) in 16HBE cells in response to serum shock synchronisation. **(L)** Heat map of the matrix coefficient correlation of each circadian clock gene and inflammatory cytokines. The stronger Pearson’s correlation coefficients (r) are represented in red. ZT= zeitgeber time. All data shown are representative data of one out of at least 3 independent experiments, Pearson’s correlation coefficient.

The altered expression of circadian clock genes has been observed in asthmatic patients with nocturnal symptoms (13). In our mouse model of asthma with circadian rhythm disorders, the circadian clock genes were also altered. Compared to the asthmatic mice in the 12 h dark/12 h light cycles, those who did not undergo these cycles exhibited an upregulation in the transcriptions of the circadian clock genes CLOCK, CRY1, CRY2, PER2, and NR1D1 (**Figures 2A–F**). We further investigated whether the upregulated circadian clock genes were associated with inflammatory cytokines. Interestingly, only IL-6 and TNF-α were most closely associated with the circadian clock genes, which suggests that an increase in IL-6 and TNF-α in asthmatic patients with nocturnal symptoms may be related to circadian clock genes (**Figure 2G**). The correlation coefficient and *p*-value have been listed in **Table S3**. Serum shock was used to stimulate the rhythmic expression of circadian clock genes in the 16HBE cells. **Figures 3A–F** shows that the transcription of CLOCK, CRY1, CRY2, PER2, and NR1D1 displayed oscillatory activity in a period of 24 ± 4 h. The transcription of IL-6 also displayed oscillatory activity that converged with the circadian clock genes (**Figure 3G**). However, serum shock did not seem to stimulate the rhythmic expression of IL-4, IL-5, IL-13, and TNF-α mRNA (**Figure 3H–K**). Correlational analyses revealed that only IL-6 and TNF-α were closely associated with the circadian clock genes following the serum shock in the 16HBE cells, which was also reflected in the asthmatic mice with circadian rhythm disorders (**Figure 3L** and **Table S4**).

### BMAL1 is the key core clock gene that drives circadian rhythms and suppresses IL-6 mRNA expression

**Figure 4:**
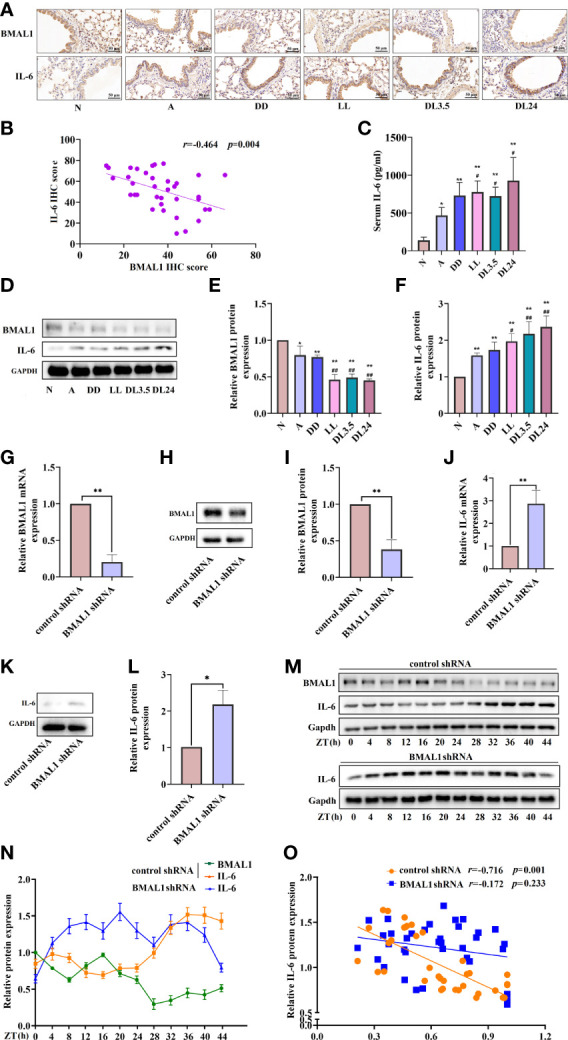
BMAL1 regulates the expression and rhythm fluctuations of IL-6. **(A)** Immunohistochemistry staining of BMAL1 and IL-6 in the mouse lung tissue of each group. Magnification: 200×. Scale bar: 50 μm. **(B)** Correlational analyses between BMAL1 and IL-6 protein expression levels in mouse lung tissue. **(C)** ELISA analysis of serum IL-6 in the mouse lung tissue of each group. **(D)** Western blot analysis of the protein levels of BMAL1 and IL-6 in the mouse lung tissue of each group. ImageJ software was used to quantify the BMAL1 **(E)** and IL-6 **(F)** bands. The mRNA **(G)** and protein **(H)** levels of BMAL1 expression in 16HBE cells with BMAL1 shRNA and control shRNA by RT-qPCR and Western blot, respectively. ImageJ software was used to quantify the BMAL1 bands **(I)**. RT-qPCR and Western blot analysis of IL-6 mRNA **(J)** and protein **(K)** levels of control shRNA and BMAL1 shRNA cells. IL-6 bands were quantitatively analysed by ImageJ software **(L)**. Western blot analysis of the protein levels of BMAL1 and IL-6 in control shRNA and BMAL1 shRNA cells in response to serum shock synchronisation **(M)**, IL-6 and BMAL1 bands were quantitatively analysed by ImageJ software **(N)**. **(O)** Correlational analyses between BMAL1 and IL-6 protein expression levels in control shRNA and BMAL1 shRNA cells. N=control group; A=OVA group; DD=OVA + all-day darkness group; LL= OVA + all-daylight; DL3.5= OVA + 3.5h dark/3.5h light group; DL24= OVA + 24h dark/24h light group; IHC, Immunohistochemistry. Data of animal experiment are presented as the mean ± SEM of 6 mice per group. Data of cell experiment shown are representative data of one out of at least 3 independent experiments. *p < 0.05, **p < 0.01, compared with the control group or control shRNA cells. ^#^ *p* < 0.05, ^##^ *p* < 0.01 compared with the OVA group. One-way ANOVA with Tukey’s multiple comparison analysis method, and independent samples t-tests, Pearson’s correlation coefficient.

BMAL1 is the principal driver of the molecular clock in mammals, and its deletion ablates rhythmic oscillatory activity (25). Therefore, we aimed to further clarify whether BMAL1 is involved in the regulation of IL-6 and whether it interferes with the correlation between other circadian clock genes and IL-6. The immunohistochemistry analysis revealed that BMAL1 and IL-6 were mainly expressed in the airway epithelial cells of mice. Compared to the normal group, BMAL1 was reduced in the lung tissue of the asthmatic mice, and it was further reduced in the non-12 h dark/12 h light cycles (**Figure 4A**). The levels of IL-6 showed an opposite trend in the lung tissue and sera of the mice (**Figures 4A, C**). BMAL1 expression was negatively correlated with IL-6 expression in mouse airway epithelial cells (*r* = –0.464, *p* = 0.004) (**Figure 4B**). Consistent with the IHC findings, Western blot analysis confirmed the protein levels of BMAL1 and IL-6 in the lungs of the mice (**Figures 4D–F**). To investigate the effects of BMAL1 knockdown on the expression of IL-6, we infected cultured 16HBE cells with a lentiviral vector encoding a shRNA targeting BMAL1 (BMAL1 shRNA) and lentiviral control (control shRNA). BMAL1 mRNA and protein levels decreased by approximately 80% and 60%, respectively, compared to cells infected with the control shRNA (**Figures 4G–I**). The knockdown of BMAL1 significantly increased the mRNA (2.8-fold increase) and protein (2.2-fold increase) levels of IL-6 in 16HBE cells (**Figures 4H–J**). After the serum shock, the protein level of BMAL1 displayed oscillatory activity with a period of 20 ± 4 h. IL-6, on the other hand, displayed opposite oscillatory activity and also showed a 20 ± 4 h cycle. The correlation analysis showed that BMAL1 was strongly negatively correlated with IL-6 and had a correlation coefficient of –0.716 (*p* = 0.001). Interestingly, the oscillatory activity of IL-6 was greatly diminished after the knockdown of BMAL1 (*r* = –0.172, *p* = 0.233) (**Figures 4K–M**).

**Figure 5:**
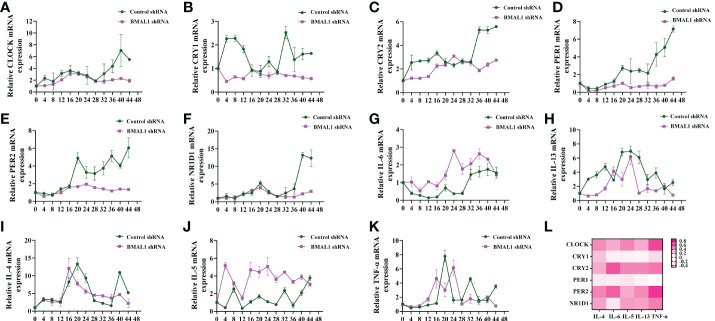
BMAL1 knockdown results in lower correlation between circadian clock genes with rhythm clock genes. **(A–F)** RT-qPCR analysis of the mRNA levels of circadian clock genes (CLOCK, CRY1, CRY2, PER1, PER2, and NR1D1) in control shRNA and BMAL1 shRNA cells in response to serum shock synchronisation. **(G–K)** RT-qPCR analysis of the mRNA levels of inflammatory cytokines (IL-6, IL-13, IL-4, IL-5, and TNF-a) in control shRNA and BMAL1 shRNA cells in response to serum shock synchronisation. **(L)** Heat map of the matrix coefficient correlation of each circadian clock gene and inflammatory cytokines in BMAL1 shRNA cells. Stronger Pearson’s correlation coefficients (r) are represented in red. ZT, zeitgeber time. All data shown are representative data of one out of at least 3 independent experiments. Pearson’s correlation coefficient.

We further explored the correlation between inflammatory factors and other rhythm clock genes after the knockdown of BMAL1. The RT-qPCR analysis showed that the knockdown of BMAL1 significantly decreased and disrupted the circadian rhythms of the negative clock genes (**Figures 5A–F**) and elevated the transcription of IL-6 and IL-5 at each time point (**Figures 5G, J**). However, there were no significant effects on the transcriptional expression of, or periodic changes in, IL-4, IL-13, and TNF-α (**Figures 5H, I, K**). Furthermore, the knockdown of BMAL1 resulted in a lower correlation between IL-6 and TNF-α with the rhythm clock genes (**Figure 5L** and **Table S5**).

### FOXA2 participates in the BMAL1 co-intervention of the circadian rhythm of IL-6

**Figure 6:**
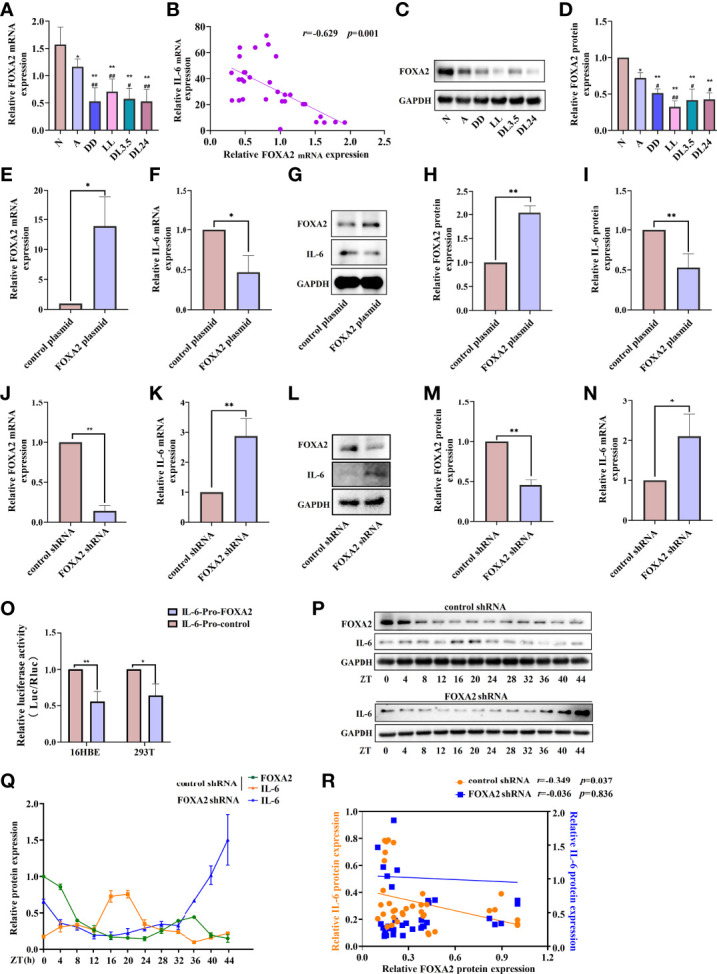
FOXA2 regulates the expression and rhythm fluctuations of IL-6. **(A)** RT-qPCR analysis of the mRNA levels of FOXA2 in lung tissue. **(B)** Correlational analyses between FOXA2 and IL-6 mRNA expression levels in lung tissue. **(C)** Western blot analysis of the protein levels of FOXA2 in mouse lung tissue of each group. ImageJ software was used to quantify FOXA2 **(D)**. RT-qPCR analysis of the mRNA levels of FOXA2 **(E)** and IL-6 **(F)** after the transfection of 16HBE cells with a FOXA2 over-expression (FOXA2 plasmid) and control plasmid. Western blot analysis of the protein levels of FOXA2 and IL-6 in 16HBE cells transfected with the FOXA2 and control plasmids **(G)**. ImageJ software was used to quantify FOXA2 **(H)** and IL-6 **(I)** bands. RT-qPCR analysis of the mRNA levels of FOXA2 **(J)** and IL-6 **(K)** expression in 16HBE cells with FOXA2 shRNA and control shRNA. Western blot analysis of the protein levels of FOXA2 and IL-6 in 16HBE cells transfected with FOXA2 shRNA and control shRNA **(L)**, ImageJ software was used to quantify FOXA2 **(M)** and IL-6 **(N)** bands. **(O)** Luciferase reporter assay analysis of the luciferase expression in the 16HBE and 293T cells transient transfection of FOXA2 and empty vector. Western blot analysis of the protein levels of FOXA2 and IL-6 in control shRNA and FOXA2 shRNA cells in response to serum shock synchronisation **(P)**, IL-6 and FOXA2 bands were quantitatively analysed by ImageJ software **(Q)**. **(R)** Correlational analyses between FOXA2 and IL-6 protein expression levels in control shRNA and FOXA2 shRNA cells. The left ordinate and the right ordinate represent the relative protein expression of IL-6 in control shRNA cells and FOXA2 shRNA cells, respectively. N=control group; A=OVA group; DD=OVA + all-day darkness group; LL= OVA + all-daylight; DL3.5= OVA + 3.5 h dark/3.5 h light group; DL24= OVA + 24 h dark/24 h light group. Data of animal experiment are presented as the mean ± SEM of 6 mice per group. Data of cell experiment shown are representative data of one out of at least 3 independent experiments. *p < 0.05, **p < 0.01, compared with the control group, control plasmid or control shRNA cells. ^#^ *p* < 0.05, ^##^ *p* < 0.01 compared with the OVA group. One-way ANOVA with Tukey’s multiple comparison analysis method, and independent samples t-tests, Pearson’s correlation coefficient.

FOXA2 is required for normal airway epithelial differentiation. Its deletion leads to Th2-mediated pulmonary inflammation during postnatal development (26). In our study, the expression of FOXA2 decreased in the lung tissue of OVA-sensitised mice. Compared to the OVA-sensitised mice in the 12 h dark/12 h light cycles, those who underwent the non-12 h dark/12 h light cycles further exhibited downregulated FOXA2 (**Figures 6A, C, D**). We also found a negative correlation between FOXA2 and IL-6 transcription in the lung tissue of mice (*r* = –0.629, *p* = 0.001) (**Figure 6B**). To further confirm the negative regulatory effect of FOXA2 on IL-6, we examined the expression of IL-6 when FOXA2 was overexpressed and knocked down using the FOXA2 plasmid and recombinant FOXA2 lentivirus vectors in 16HBE cells. Transfected 16HBE cells with a FOXA2 plasmid and FOXA2 mRNA and protein levels increased by approximately 14-fold and 2-fold, respectively, while IL-6 decreased by approximately 50% compared to cells infected with the control plasmid (**Figures 6E–I**). In contrast, **Figures 6J–N** shows that the knockdown of FOXA2 significantly improved the expression of IL-6. To demonstrate the function of FOXA2 in IL-6 regulation, co-transfection with the IL-6-luc and FOXA2 plasmids was performed. The transient transfection of FOXA2 significantly decreased luciferase expression compared with the empty vector in the 16HBE and 293T cells (**Figure 6O**). After serum shock, the protein level of FOXA2 displayed oscillatory activity, while IL-6 displayed an opposite oscillatory activity with a 24 ± 4 h cycle. The correlation analysis showed that the expression of FOXA2 was significantly negatively correlated with IL-6 expression (*r* = –0.349, *p* = 0.037). Nevertheless, the oscillatory activity of IL-6 was greatly diminished when FOXA2 was knocked down (*r* = –0.036, *p* = 0.836) (**Figures 6P–R**).

**Figure 7:**
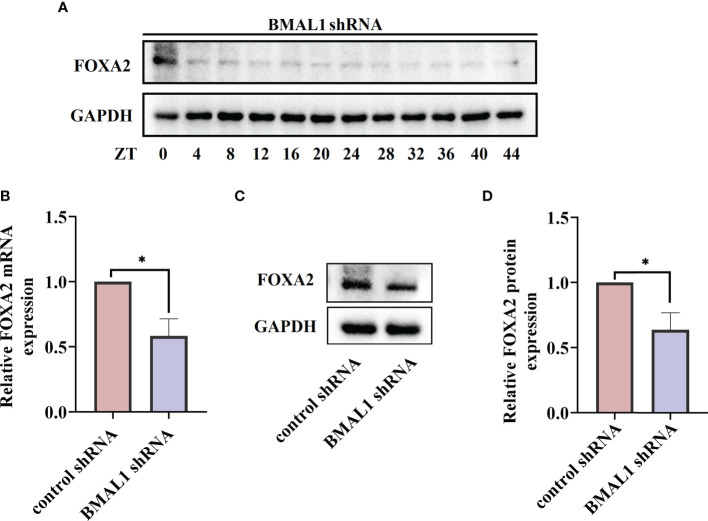
BMAL1 regulates the expression and rhythm fluctuations of FOXA2 in 16HBE cells. **(A)** Western blot analysis of the protein levels of FOXA2 in BMAL1 shRNA cells in response to serum shock synchronisation. The mRNA **(B)** and protein **(C)** levels of FOXA2 expression in 16HBE cells with BMAL1 shRNA constructs and control shRNA by RT-qPCR and Western blot, respectively. ImageJ software was used to quantify FOXA2 bands **(D)**. by RT-qPCR and Western blot, respectively. ImageJ software was used to quantify BMAL1 bands (g). ZT, zeitgeber time. All data shown are representative data of one out of at least 3 independent experiments, *p < 0.05 compared with control shRNA cells, independent samples t-tests.

**Figure 8:**
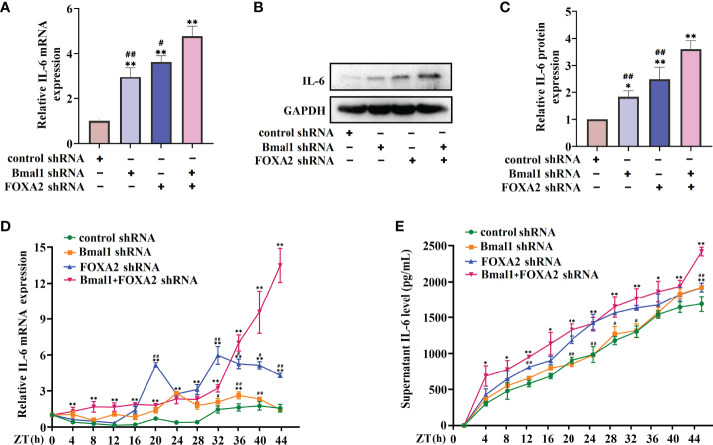
Double FOXA2 and BMAL1 stable depletion causes a significant increase of IL-6. The mRNA **(A)** and protein **(B)** levels of IL-6 in 16HBE cells transfected with control shRNA, FOXA2 shRNA, BMAL1 shRNA, and FOXA2+BMAL1 shRNA by RT-qPCR and Western blot, respectively. ImageJ software was used to quantify the IL-6 bands **(C)**. RT-qPCR **(D)** and ELISA **(E)** analysis of mRNA and secretion levels of IL-6 in 16HBE cells transfected with control shRNA, FOXA2 shRNA, BMAL1 shRNA, and FOXA2+BMAL1 shRNA cells in response to serum shock synchronisation. ZT, zeitgeber time. All data shown are representative data of one out of at least 3 independent experiments, *p < 0.05, **p < 0.01 compared with control shRNA cells. ^#^ *p* < 0.05, ^##^ *p* < 0.01 compared with FOXA2+BMAL1 shRNA cells. One-way ANOVA with Tukey’s multiple comparison analysis method, and independent samples t-tests.

These results suggest that FOXA2 may function as a negative regulator in the regulation of IL-6 by rhythm clock genes. We further explored the expression of FOXA2 in BMAL1-knockdown 16HBE cells. The oscillatory activity of FOXA2 greatly disappeared after the serum shock in BMAL1-knockdown 16HBE cells (**Figure 7A**). A significant decrease in the expressions of FOXA2 mRNA and protein were observed after the knockdown of BMAL1 (**Figures 7B–D**). In addition, we found that IL-6 had a weakened increase by transfecting the FOXA2 overexpression plasmid in BMAL1-knockdown 16HBE cells (**Figure S2**). Subsequently, the 16HBE cells were subjected to the knockdown of FOXA2 and BMAL1. The double knockdown of FOXA2 and BMAL1 caused a significant increase in IL-6 levels (**Figures 8A–C**). Furthermore, after the serum shock, the IL-6 transcription and secretion levels were significantly upregulated in BMAL1- and FOXA2-knockdown 16HBE cells compared to single FOXA2 or BMAL1 stable depletion cells (**Figures 8D, E**).

## Discussion

The master pacemaker of the mammalian circadian clock is located in the hypothalamic suprachiasmatic nucleus (SCN). Intrinsically photosensitive retinal ganglion cells (ipRGCs) that respond to the lighting cycles and pass this information on to the SCN involve a complete network of clock genes (27). Complexes of the BMAL1-CLOCK transcription factor bind to cis-acting elements known as the E-box and activate the transcription of the PER1, PER2, CRY1, CRY2, NR1D1, and NR1D2 clock genes (28). The CRY and PER proteins form heterodimers and translocate back into the nucleus, inhibiting transcriptional activation by BMAL1-CLOCK to form the core negative feedback loop. Accompanied by the degradation of PER and CRY proteins, the repression of BMAL1 is gradually released, and a new PER/CRY transcription cycle is subsequently formed (29). BMAL1-CLOCK also forms a second core loop involving the NR1D1/2 nuclear receptor genes, which allows for the rhythmic expression of BMAL1 (30).

Light and darkness are the primary regulators of the circadian clock in mammals. With urbanisation and the rapid development of lighting systems, long-term exposure to artificial light leads to the breakdown of the circadian rhythm balance in the body and contributes to sleep disorders, gastrointestinal system disorders, accelerated aging, neurodegenerative disorders, obesity, cancer, and worsening asthmatic severity (31–33). Although the mechanisms by which circadian clocks contribute to these diseases are unclear, the diseases share common mechanisms, such as inflammation, which can further disrupt circadian rhythms (34). Mice carrying a clock gene mutation or deletion have exacerbated airway inflammation conditions. For example, mice lacking BMAL1 in myeloid cells have been found with increased lung inflammation, eosinophil infiltration, and inflammatory cytokines in their lungs and sera (35). The deletion of BMAL1 or disruption of the circadian clock environment exacerbates acute viral bronchiolitis caused by the Sendai and influenza A viruses in mice (36). Chronic jetlag has also been found to affect tumour-bearing mice and increases inflammation in the hypothalamus and liver, suggesting that the inflammatory signatures in the body can be further magnified by circadian disruption (33). Therefore, asthma with circadian rhythm disturbances may amplify the inflammatory signatures of the body, leading to inadequate asthma control and triggering of attacks at night.

Abnormal 12 h dark/12 h light cycles have been used to induce animal models with circadian rhythm disturbances. Adult male rats exposed to prolonged light for 12 consecutive weeks showed the upregulated relative mRNA expression of PER2, CRY2, and NR1D1 and downregulated mRNA expression of PER1, CRY1, BMAL1, and CLOCK (18). The expression of PER1 and PER2 in rat livers lost rhythmicity in a constant light environment, while the expression of NR1D1 maintained rhythmicity (37). Asthmatics showed increased rhythmic gene amplitudes (38). Furthermore, the circadian oscillation of BMAL1 content in the SCN of rats in constant darkness was stronger, and the peak/trough ratios were higher than those of rats undergoing 12 h dark/12 h light cycles (39). Although a consensus has been reached on the role of rhythmic disturbances in nocturnal asthma attacks, animal models of rhythmic disturbances are not the same (35). BMAL1-deficient mice have been used for rhythm research to show the disappearance of rhythmic oscillations, which is inconsistent with asthmatic rhythm gene expression patterns (40). In this study, we used four abnormal 12 h dark/12 h light cycles to induce rhythm disturbances in OVA-challenged mice, which showed varying degrees of downregulated BMAL1 expression and upregulated IL-6. This may explain the concomitant increase in IL-6 in patients with nocturnal asthma.

Nocturnal asthma symptoms are associated with inflammation in the lung tissue. Previous studies have confirmed an increase in inflammatory cell populations and cytokines in the BALF of patients with nocturnal asthma (41–43). This inflammatory response, its induced airway epithelial damage, and small airway spasms may be important factors in the etiology of nocturnal asthma. By comparing the blood samples of asthmatic patients with and without nocturnal symptoms between 4–5 A.M., we found that the expression of IL-6 in patients with nocturnal asthma attacks was significantly higher than that in asthmatic patients without nocturnal symptoms. TNF-α also presented a significant difference in asthmatic patients with and without nocturnal symptoms, but it did not show a significant difference after adjustment by the Benjamini-Hochberg correction method for *p*-values. These results were the same as those obtained by Tom et al. (42), and may be related to insufficient sample size. The relationship between rhythmic genes and inflammation involves a complex traffic network. The dysregulation of the clock leads to dysregulated inflammation. Since inflammation can directly affect circadian rhythmicity, this constitutes a vicious cycle (44). We hope to use IL-6 as an example to provide research ideas for the study of circadian clock genes that regulate changes in airway inflammation.

The transcription factor BMAL1, an essential component of the molecular clock, regulates the upregulation of IL-6 in lipopolysaccharide-exposed microglia and plays a role in microglial inflammatory responses (45). The loss of BMAL1, or jetlag-sensitised mice, acts on the NF-κB/NLRP3 axis to promote inflammation, which exacerbates *Propionibacterium acnes*-induced skin inflammation (46). In addition, BMAL1 plays an important role in critical limb ischemia by activating IL-10, which transcriptionally suppresses inflammation and promotes angiogenesis *via* the transcriptional regulation of vascular endothelial growth factor expression (47). However, the role of BMAL1 in regulating lung inflammation remains unclear. Here, we found that the loss of the BMAL1 gene can lead to increased IL-6 expression and loss of rhythm in airway epithelial cells while reducing the correlation between IL-6 and other negatively regulated circadian clock genes. This suggests that the disorder of circadian clock genes and low expression of BMAL1 may lead to an increase in cytokines such as IL-6 and induce nocturnal asthma symptoms.

The transcription factor FOXA2, which belongs to the forkhead family, is involved in the development of lung tissues and is highly expressed in the airway epithelial cells of mature lungs. The deletion of FOXA2 has been found to cause goblet cell metaplasia and Th2-mediated lung inflammation in respiratory epithelia (48, 49). FOXA2 is expressed at low levels in asthmatic patients and is negatively correlated with the expression of mucins, such as MUC5AC and CLCA1 (48). FOXA2 is negatively correlated with IL-6 in patients with chronic rhinosinusitis. Increasing the expression of FOXA2 inhibits IL-6-induced MUC5AC production (50). The secretion of TNF-α, IL-1β, and IL-6 is inhibited in MLE-12 cells with low FOXA2 expression, and oxidative stress-induced apoptosis is also reduced (51). We also found a negative correlation between FOXA2 and IL-6 in OVA-challenged mice and the 16HBE cell serum shock model. Interestingly, FOXA2 also displayed oscillatory activity in serum shock, suggesting that FOXA2 may be involved in the transcriptional regulation of IL-6 by circadian clock genes.

**Figure 9:**
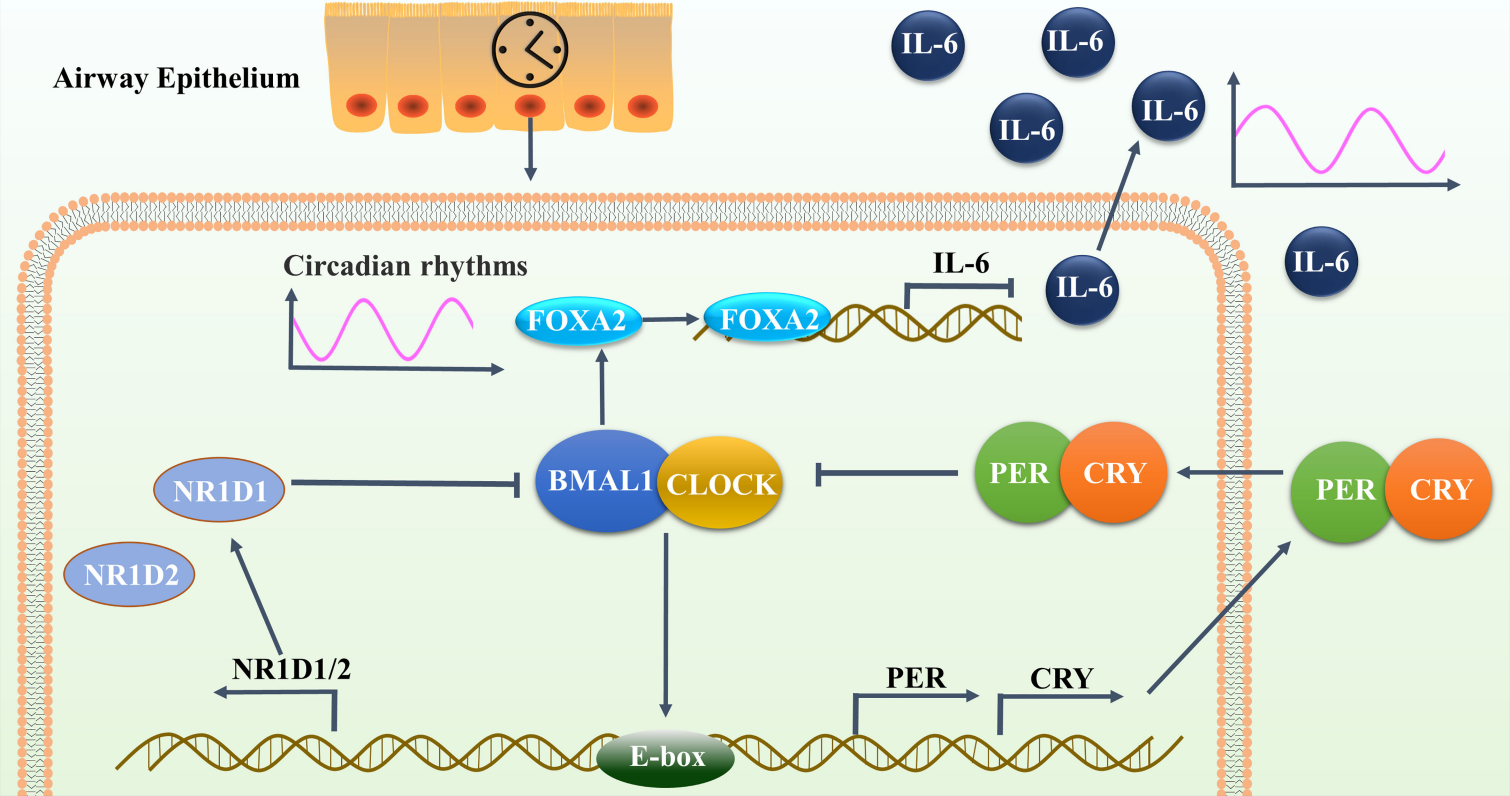
BMAL1 inhibited the expression and reduced the oscillation of IL-6 in airway epithelial cells by regulating the transcription factor FOXA2. This autoregulatory feedback loop cycles between the CLOCK/BMAL1 transcriptional activator complex and its repressors PER/CRY and NR1D1 to constitute the molecular clock oscillator in the airway epithelium, that drives the oscillatory activity of FOXA2. FOXA2 could bind to the promoter region of IL-6 and down-regulate its expression, resulting in periodic oscillations in the secretion level of IL-6.

The molecular clock also plays an important regulatory role in mammalian embryonic development. The disruption of BMAL1 in mammals during embryonic development can lead to a reduced lifespan, locomotor activity level, body weight, and fertility (52). The activation of FOXA2 is severely hindered during the differentiation of BMAL1-knockdown mouse embryonic stem cells (53). Differentiated human colon adenocarcinoma cells have been found to correlate with decreased levels of FOXA2 upon treatment with shBMAL1. In turn, BMAL1 overexpression has no effect on FOXA2 expression (54). Here, we observed the lower expression and reduced rhythms of FOXA2 in 16HBE cells after BMAL1 suppression. We further noted the synergistic inhibitory effects of the double knockdown of FOXA2 and BMAL1 on the expression and oscillation of IL-6 in 16HBE cells. This study confirmed that BMAL1 inhibits the expression and oscillations of IL-6 in airway epithelial cells by regulating the transcription factor FOXA2 (**Figure 9**).

To the best of our knowledge, our findings are the first to demonstrate that there is a correlation between the increase in IL-6 inflammatory factors and rhythm clock genes in patients with nocturnal asthma. Mechanistic investigations supported the activation of BMAL1/FOXA2 pathway in reducing the secretion of IL-6 in airway epithelial cells. Targeting the BMAL1/FOXA2 pathway therefore represents a potential therapeutic option for inhibiting the increase of airway inflammation at night in asthmatic patients with nocturnal symptoms.

## Data availability statement

The original contributions presented in the study are included in the article/**Supplementary Material**. Further inquiries can be directed to the corresponding authors.

## Ethics statement

This study was approved by the Ethics Committee of the Affiliated Hospital of Nanjing University of Chinese Medicine (approval number 2019NL-024-02). All patients provided informed consent. The patients/participants provided their written informed consent to participate in this study. All of the protocols involving animal use were approved by the Animal Ethics Committee of the Affiliated Hospital of Nanjing University of Traditional Chinese Medicine (NO:2021 DW-07-02).

## Author contributions

LT, XZ, XLS designed the experiments. PH, XZ participated in the collection and statistics of clinical cases. XHS, JJ, BW verified the underlying data. XHS, XNZ, and PH performed the animal model. LT and XHS completed the cell serum shock test. BW, XNZ and JJ processed the BALF and mouse lung tissue samples. LT and HZ carried out the RT-qPCR and IHC analysis. LT wrote the manuscript. All authors contributed to the article and approved the submitted version.

## Funding

This work was financially supported by National Natural Science Foundation of China 82004265, 82004304, and General project of Jiangsu Natural Science Foundation BK20211391, and Graduate Research and Practice Innovation Plan of Graduate Education Innovation Project in Jiangsu Province SJCX21-0742. 2020 Nanjing Traditional Chinese Medicine Young Talents Training Project NJSZYYQNRC-2020-ZH.

## Acknowledgments

The authors sincerely acknowledge the support of the Central Laboratory of Jiangsu Hospital of Traditional Chinese Medicine for providing the experimental platform and guidance.

## Conflict of interest

The authors declare that the research was conducted in the absence of any commercial or financial relationships that could be construed as a potential conflict of interest.

## Publisher’s note

All claims expressed in this article are solely those of the authors and do not necessarily represent those of their affiliated organizations, or those of the publisher, the editors and the reviewers. Any product that may be evaluated in this article, or claim that may be made by its manufacturer, is not guaranteed or endorsed by the publisher.

## Supplementary material

Supplementary Figure 1 Flowchart of creating light-induced disturbances of rhythm in OVA-challenged mice. i.p., intraperitoneal; i.n.= intranasal; N, control group; A=OVA group; DD=OVA + all-day darkness group; LL= OVA + all-daylight; DL3.5= OVA + 3.5 h dark/3.5 h light group; DL24= OVA + 24 h dark/24 h light group.

Supplementary Figure 2 IL-6 had a weakened increase by transfecting the FOXA2 overexpression plasmid in BMAL1-knockdown 16HBE cells. **(A)** Western blot analysis of the protein levels of IL-6 after the transfection of BMAL1-knockdown 16HBE cells with FOXA2 plasmid. **(B)** ImageJ software was used to quantify IL-6 bands. All data shown are representative data of one out of at least 3 independent experiments, **p* < 0.05 compared with control shRNA cells. #*p* < 0.05 compared with BMAL1 shRNA cells. One-way ANOVA with Tukey’s multiple comparison analysis method.

Supplementary Table 1 The target sequences of shRNAs.

Supplementary Table 2 Sequences of primers used.

Supplementary Table 3 Pearson’s correlation coefficient for the concentrations of circadian clock genes and inflammatory cytokines in the mice lung tissues. Pearson’s correlation coefficient was used to measure the strength of the relationship between the two variables. **p* < 0.05, ***p* < 0.01.

Supplementary Table 4 Pearson’s correlation coefficient for the concentrations of circadian clock genes and inflammatory cytokines in 16HBE cells in response to serum shock synchronisation. Pearson’s correlation coefficient was used to measure the strength of the relationship between the two variables. **p* < 0.05, ***p* < 0.01.

Supplementary Table 5 Pearson’s correlation coefficient for the concentrations of circadian clock genes and inflammatory cytokines in BMAL1 knockdown 16HBE cells in response to serum shock synchronisation. Pearson’s correlation coefficient was used to measure the strength of the relationship between the two variables. **p* < 0.05, ***p* < 0.01.
