# Supplementary figures and images for: BMAL1/FOXA2-induced rhythmic fluctuations in IL-6 contribute to nocturnal asthma attacks
Source: Front Immunol. 2022 Nov 25;13:947067. doi: 10.3389/fimmu.2022.947067 (PMC9732258; doi:10.3389/fimmu.2022.947067)

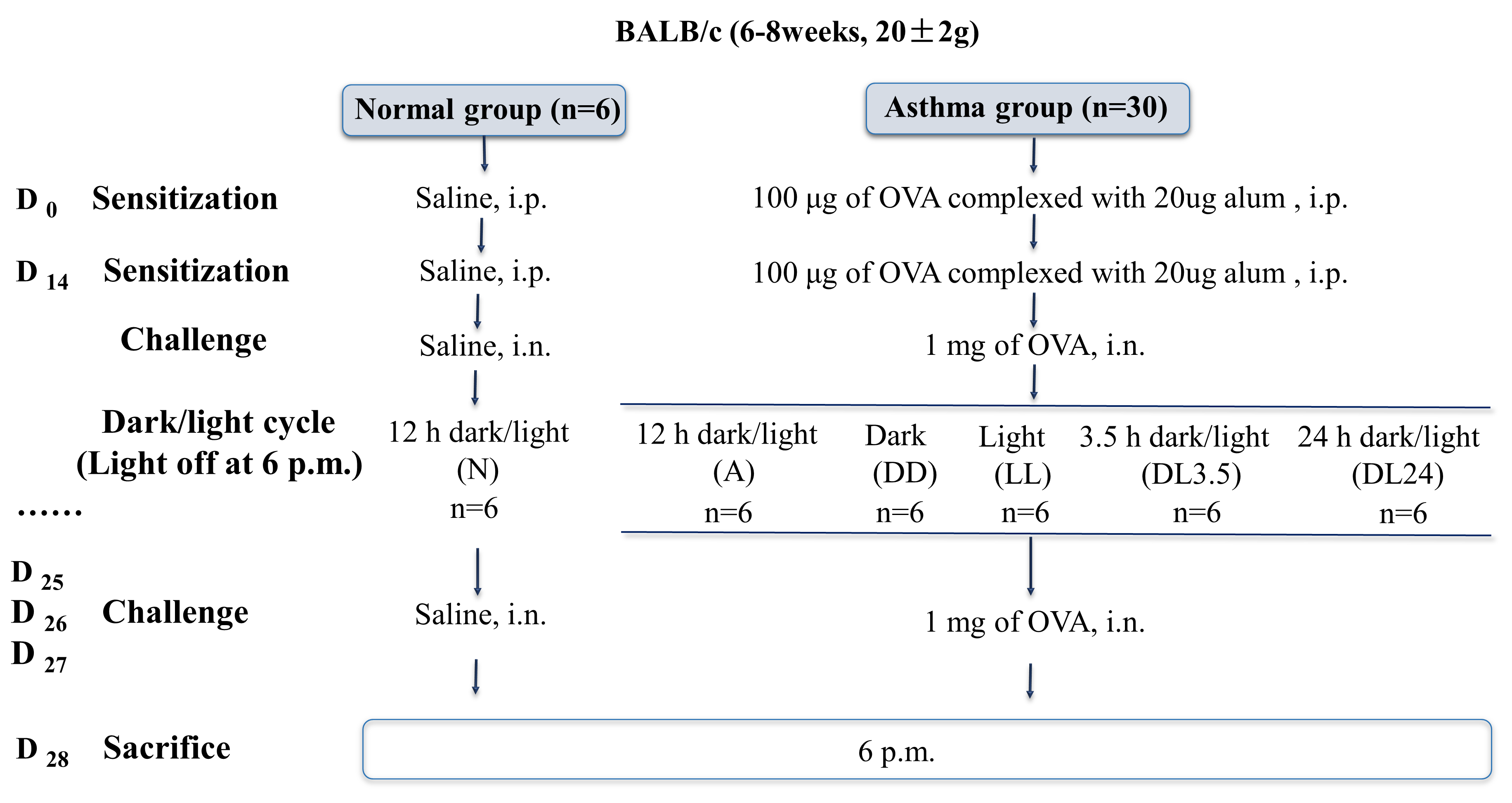

Supplement: Supplementary Figure 1 — Flowchart of creating light-induced disturbances of rhythm in OVA-challenged mice. i.p., intraperitoneal; i.n.= intranasal; N, control group; A=OVA group; DD=OVA + all-day darkness group; LL= OVA + all-daylight; DL3.5= OVA + 3.5 h dark/3.5 h light group; DL24= OVA + 24 h dark/24 h light group. [file Image_1.tif]

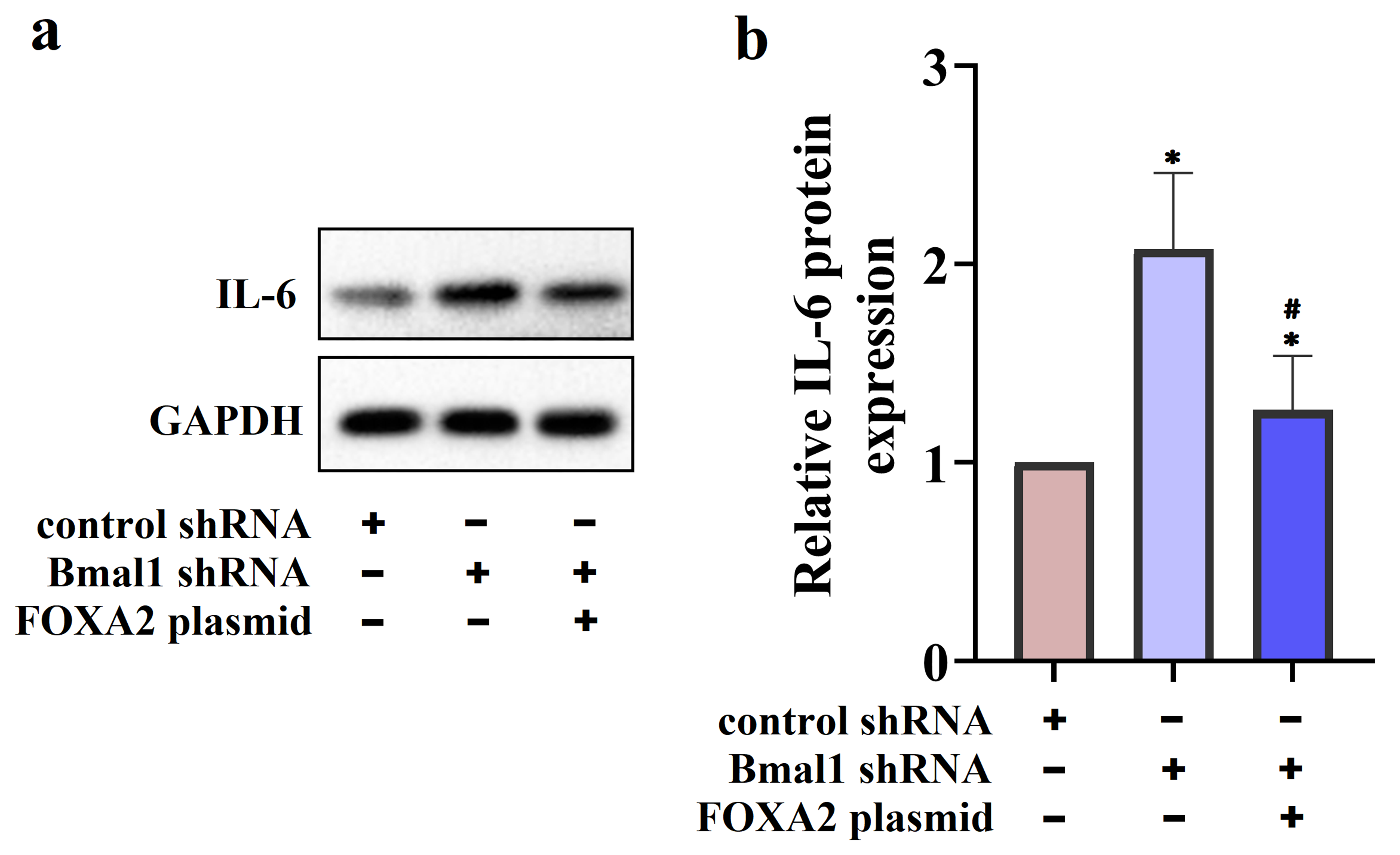

Supplement: Supplementary Figure 2 — IL-6 had a weakened increase by transfecting the FOXA2 overexpression plasmid in BMAL1-knockdown 16HBE cells. (A) Western blot analysis of the protein levels of IL-6 after the transfection of BMAL1-knockdown 16HBE cells with FOXA2 plasmid. (B) ImageJ software was used to quantify IL-6 bands. All data shown are representative data of one out of at least 3 independent experiments, *p < 0.05 compared with control shRNA cells. #p < 0.05 compared with BMAL1 shRNA cells. One-way ANOVA with Tukey’s multiple comparison analysis method. [file Image_2.tif]
